# Genomic profile of SARS-CoV-2 Omicron variant and its correlation with disease severity in Rajasthan

**DOI:** 10.3389/fmed.2022.888408

**Published:** 2022-09-23

**Authors:** Ravi P. Sharma, Swati Gautam, Pratibha Sharma, Ruchi Singh, Himanshu Sharma, Dinesh Parsoya, Farah Deeba, Neha Bhomia, Nita Pal, Varsha Potdar, Pragya D. Yadav, Nivedita Gupta, Sudhir Bhandari, Abhinendra Kumar, Yash Joshi, Priyanka Pandit, Bharti Malhotra

**Affiliations:** ^1^Medical and Health Rajasthan, Jaipur, India; ^2^Department of Microbiology, Sawai Man Singh Medical College, Jaipur, India; ^3^Virology Department, National Institute of Virology (ICMR), Pune, India; ^4^Virology Department, Indian Council of Medical Research (ICMR), New Delhi, India

**Keywords:** SARS-CoV-2, NGS, Omicron, pandemic, BA.2, BA.1

## Abstract

**Background:**

Omicron, a new variant of Severe Acute Respiratory Syndrome-Coronavirus 2 (SARS-CoV-2), was first detected in November 2021. This was believed to be highly transmissible and was reported to evade immunity. As a result, an urgent need was felt to screen all positive samples so as to rapidly identify Omicron cases and isolate them to prevent the spread of infection. Genomic surveillance of SARS-CoV-2 was planned to correlate disease severity with the genomic profile.

**Methods:**

All the SARS-CoV-2 positive cases detected in the state of Rajasthan were sent to our Lab. Samples received from 24 November 2021 to 4 January 2022 were selected for Next-Generation Sequencing (NGS). Processing was done as per protocol on the Ion Torrent S5 System for 1,210 samples and bioinformatics analysis was done.

**Results:**

Among the 1,210 samples tested, 762 (62.9%) were Delta/Delta-like and other lineages, 291 (24%) were Omicron, and 157 (12.9%) were invalid or repeat samples. Within a month, the proportion of Delta and other variants was reversed, 6% Omicron became 81%, and Delta and other variants became 19%, initially all Omicron cases were seen in international travelers and their contacts but soon community transmission was seen. The majority of patients with Omicron were asymptomatic (56.7%) or had mild disease (33%), 9.2% had moderate symptoms, and two (0.7%) had severe disease requiring hospitalization, of which one (0.3%) died and the rest were (99.7%) recovered. History of vaccination was seen in 81.1%, of the previous infection in 43.2% of cases. Among the Omicron cases, BA.1 (62.8%) was the predominant lineage followed by BA.2 (23.7%) and B.1.529 (13.4%), rising trends were seen initially for BA.1 and later for BA.2 also. Although 8.9% of patients with Delta lineage during that period were hospitalized, 7.2% required oxygen, and 0.9% died. To conclude, the community spread of Omicron occurred in a short time and became the predominant circulating variant; BA.1 was the predominant lineage detected. Most of the cases with Omicron were asymptomatic or had mild disease, and the mortality rate was very low as compared to Delta and other lineages.

## Background

On 24 November 2021, B.1.1.529, a new variant of Severe Acute Respiratory Syndrome-Coronavirus 2 (SARS-CoV-2), was reported to the World Health Organization (WHO). This variant was first detected in Botswana on 11 November 2021 and on 14 November 2021 in South Africa, which was later termed Omicron by WHO and declared a variant of concern (VOC) eventually^1^. Omicron (21 M) or the Pango lineage B.1.1.529 includes 21K Omicron (BA.1), its sister clade 21L Omicron (BA.2), and other diverse Omicron sequences. 21L and 21K share 38 mutations but 21L has additional 27 mutations (with12 unique mutations), and 21 K has 20 more (6 unique deletion/mutation), 21L lacks the SH69 and Sv70 deletions, which lead to S Gene Target Failure (SGTF) that has been used a proxy marker for Omicron in TaqPath PCR Kits (1).

Many studies have reported that Omicron spreads faster than the Delta variant, up to 3.31 times faster than the Delta variant (2). It can evade the immunity provided by natural infection and vaccination due to the mutations, which are known to increase transmission, immune escape, and enhance binding affinity (3). The preliminary data on Omicron suggest that the illness caused may be asymptomatic to mild disease. However, the severity of the disease due to the Omicron variant remains questionable as many factors, such as immune status, age, co-morbid conditions, etc. may affect it and vary in different regions (4). Omicron has been reported from various countries and now from many states of India too. It is predicted that very soon it will take over the Delta strain and become the dominant strain. Rajasthan is the largest state of India with a 342,239 square kilometers area and a 78.23 million population. Rajasthan witnessed a very high number of SARS-CoV-2 cases during the second pandemic peak, on 6 May 2021, 7,532 new cases were reported and as per Integrated Diseases Surveillance Program (IDSP) data, 17.7% positivity rate, and 1.2% death rate (4,146 deaths) were seen in May 2021. It has been observed that there is a correlation between population density and basic reproductive number (R_0_) of SARS-CoV-2 and disease transmission (5). To contain the infection, it is important to isolate the individuals who have been infected with SARS-CoV-2. It is important to carry out genomic surveillance for the early detection of new variants for effective control and treatment.

The objective of the study was to carry out the genomic surveillance of SARS-CoV-2 and correlate it with the severity of disease in patients with Omicron and other variants.

## Methods

### Study design and sample collection

Genomic surveillance of SARS-CoV-2 from all positive cases in Rajasthan state, especially the foreign travelers and their contacts, was initiated by the Government of Rajasthan at Sawai Man Singh Medical College (SMSMC) Jaipur. Jaipur lab was designated as the satellite lab of the Indian SARS-CoV-2 Genomics Consortium (INSACOG), and the National Institute of Virology (NIV) Pune (national reference laboratory for virology and SARS-CoV-2 testing in India) was the hub lab for SMSMC. The fund for genomic surveillance was provided by the Government of Rajasthan. The study was approved by the ethics committee of SMSMC, Jaipur (ref. no. 299/MC/EC/2022). All SARS-CoV-2 positive samples from all the 33 districts of Rajasthan were sent to SMSMC for gene sequencing through the state IDSP team along with clinical details and vaccination status. The international travelers were tested on priority. Samples received from 24 November 2021 to 4 January 2022 were included in the study.

### Nucleic acid extraction and real-time PCR

Nasopharyngeal/throat swab specimens, which were collected in Viral Transport Medium (VTM) from all over Rajasthan, from positive patients with SARS-CoV-2 were received at SMSMC, Jaipur for Next-Generation Sequencing (NGS). Samples received from 24 November 2021 to 4 January 2022 were included in this study. Nucleic acid extraction was done on an automated extraction system, NucliSENS easyMAG (BioMérieux, France) using 400 μl VTM. Samples were retested for SARS-CoV-2 using TRUPCR (3B BlackBio Biotech, India) real-time reverse transcriptase PCR (RT-PCR) kit in our lab for checking the cycle threshold (Ct) value of the sample.

### Genome sequencing

In total, 1,210 samples, having high viral load (Ct < 25 for E and open reading frame (ORF) gene), were selected for genome sequencing (6). Briefly, quantification of extracted RNA was done using a Qubit HSRNA Kit (Life Technologies, USA). Superscript VILO Reverse Transcriptase Kit (Invitrogen, USA) was used for cDNA synthesis. Library preparation was done using Ion AmpliSeq Library Plus Kit (Life Technologies) and Ion AmpliSeq SARS-CoV-2 Research Assay Panel (Life Technologies), which consists of two primer pools that target amplicons ranging from 125 to 275 bp in length for complete coverage of over 99% of viral genome and variants. Briefly, two pools of amplicons prepared from cDNA were combined to make a single amplicon pool, which was then partially digested with FuPa reagent followed by ligation of specific barcode adaptors. Prepared libraries were purified and finally amplified before library quantification using a Qubit dsDNA High Sensitivity Kit on Qubit 2.0 Fluorometer (Thermo Fisher Scientific, Waltham, MA, USA). Libraries having concentrations <300 ng/ml were rejected. The libraries were diluted to 20 pM and multiple diluted libraries were pooled in equal volumes before running on the Ion One Touch 2 Instrument, which prepares template-positive ion sphere particles (ISPs) containing clonally amplified DNA, using the Ion 530–OT2 Kit. The template-positive ISPs were enriched with the Ion One Touch ES instrument and were loaded on an Ion 530 Chip. The loaded chips were sequenced on the Ion S5 Next-Generation Sequencing (NGS).

### NGS data quality check and analysis

Base calling and data processing were done by using various plugins, i.e., Coverage Analysis, SARS-CoV-2 Variant Caller, Generate Consensus, and SARS-CoV-2 Lineage ID using the Torrent Suite software v5.12.0 (Thermo Fisher Scientific, USA). Torrent Mapping Alignment Program (TMAP) in Torrent Suite Software was used for the alignment of reads with the reference genome of SARS-CoV-2 (Gen Bank accession NC_045512.2). The process involved aligning reads produced by the pipeline to the SARS-CoV-2 reference sequence and extracting metrics from those alignments. The output of the alignment process was in a Binary Alignment Map **(**BAM) file. The BAM file included alignment of all reads, including the unmapped reads, with exactly one mapping per read. The number of called bases with a predicted quality of Q20 was reported. The criteria to define a valid sequence were the number of reads higher than 1 million and <1% of unknown nucleotides (N) in the sequence. A total of 1,053 good-quality sequences were submitted to the Global Initiative on Sharing All Influenza Data (GISAID) EpiCoV repository.

### Construction of phylogenetic tree of Omicron sequences

Fast alignment sequence test for application (FASTA) sequences generated from generate consensus plugin was downloaded and these were aligned with Wuhan-Hu-1/2019 (Genbank: MN908947) as a reference, and the sequences were downloaded from GISAID (7) using Nextclade (https://clades.Nextstrain.org). Nextclade carries out different processes, such as sequence alignment (pairwise alignment using a variation of the Smith-Waterman algorithm), clade assignment, and phylogenetic placement of these sequences, which can be visualized and/or downloaded. The phylogenetic tree was constructed, downloaded, and visualized using Nextstrain Auspice (accessed on 05 February 2022) web software.

### Mutation analysis of Omicron sequences

Mutation analysis of Omicron sequences was done at NIV, Pune. The data generated through NGS were analyzed by using software CLC Genomics version 21.0.4, while the GraphPad (PRISM 9.2.0) was used to construct a heat map of Omicron variant analysis.

### Statistical analysis

Metadata of the patients was noted in terms of age, gender, VOC type, vaccination status, and clinical outcomes (hospitalization, oxygen requirement, and death). The data were analyzed and correlated with the results of whole genome sequencing. Clinical characteristics and outcomes were reported as either counts or percentages and compared between patients with Omicron variant vs. Delta variant. Comparisons for dichotomous variables were done by chi-square test (Fisher's exact test for counts <5). All statistical tests were two-sided, and *p* < 0.05 were considered statistically significant.

## Results

### Baseline characteristics

Among the 1,210 samples tested, 762 (62.9%) were Delta/Delta-like and other lineages, 291 (24%) were Omicron, and 157 (12.9%) were invalid or repeat samples. Among the 291 Omicron cases, higher positivity was seen in male patients (56.7%) than in female patients (43.2%). Moreover, the highest positivity (68%) was seen in the 19–59 age group. In total, 50 (17.18%) of Omicron cases were ≤18 years old and 82.82% were >18 years old. In addition, in the pediatric age group, 17.10% positivity was observed. Among the 762 Delta/Delta-like and other variants, 480 cases (62.99%) were male patients, and 282 (37.01%) were female patients; 82 (10.76%) cases were ≤18 years old and 680 (89.34%) were >18 years old.

### Family cluster and history of contact or travel in Omicron cases

The first nine Omicron positive cases were seen in a family with a history of travel to South Africa and their close contacts. The family of four international travelers reported negative for SARS-CoV-2 in South Africa and in Dubai before arriving in India. On reaching Rajasthan, they visited their relatives. One of the local relatives developed mild symptoms and was found positive, which led to the testing of the family and after which the other four contacts came positive (a total of 5). On tracing their contact history, the international travelers were traced and were found to be positive. Eventually, other members of the local family and their driver with his family also became positive for Omicron, thus affecting 19 persons in the cluster. Earlier samples were of B.1.1.529 lineage and later cases were found to be of BA.1. Another big cluster of nine patients from Jaipur district jail was found to be BA.2 positive and had mild symptoms. Sequentially, a total of 1,053 SARS-CoV-2 RT-PCR positive samples were sequenced among which 291 (27.64%) cases were identified as Omicron variants. Among the 291 Omicron cases, 45 (15.4%) had a history of international travel, 33 (11.3%) national travel, and 68 (23.4%) of known positive cases. No history of contact or travel was obtained in 145 (49.8%) patients (Table 1).

**Table 1:** International travel history in Omicron cases.

**District**	**NO**	**Status of vaccination**				**Travel History**			
	**No**	**Partially**	**Fully**	**Not eligible**	**Unvaccinated**	**International**	**National**	**Contact history**	**Others**
		**No**	**No**	**No**	**No**	**No**	**No**	**No**	**No**
Jaipur	206	10	173	15	8	23	24	42	117
	**%**	**4.8**	**83.9**	**7.3**	**3.8**	**11.1**	**11.6**	**20.3**	**56.8**
Ajmer	20	0	16	4	0	9	2	6	3
	**%**	**0.0**	**80.0**	**20.0**	**0.0**	**45.0**	**10.0**	**30.0**	**15.0**
Udaipur	9	0	9	0	0	2	0	4	3
	**%**	**0.0**	**100**	**0.0**	**0.0**	**22.2**	**0.0**	**44.4**	**33.3**
Sikar	6	1	3	1	1	0	0	5	1
	**%**	**16.6**	**50.0**	**16.6**	**16.6**	**0.0**	**0.0**	**83.3**	**16.6**
Bhilwara	7	1	3	2	1	2	1	0	4
	**%**	**14.3**	**42.8**	**28.5**	**14.3**	**28.5**	**14.3**	**0.0**	**57.1**
Jodhpur	3	0	3	0	0	2	0	1	0
	**%**	**0.0**	**100.0**	**0.0**	**0.0**	**66.6**	**0.0**	**33.3**	**0.0**
Alwar	8	1	6	0	1	1	1	1	5
	**%**	**12.5**	**75.0**	**0.0**	**12.5**	**12.5**	**12.5**	**12.5**	**62.5**
Bikaner	3	0	2	1	0	0	0	0	3
	**%**	**0.0**	**66.6**	**33.3**	**0.0**	**0.0**	**0.0**	**0.0**	**100.0**
Pratapgarh	7	0	6	1	0	3	0	4	00.0
	**%**	**0.0**	**85.7**	**14.3**	**0.0**	**42.8**	**0.0**	**57.1**	**0**
Sirohi	3	1	1	1	0	3	0	0	0
	**%**	**33.3**	**33.3**	**33.3**	**0.0**	**100**	**0.0**	**0.0**	**0.0**
Bharatpur	2	0	1	0	1	0	0	0	2
	**%**	**0.0**	**50.0**	**0.0**	**50.0**	**0.0**	**0.0**	**0.0**	**100.0**
Hanumangarh	1	0	0	0	1	0	0	0	1
	**%**	**0.0**	**0.0**	**0.0**	**100**	**0.0**	**0.0**	**0.0**	**100.0**
Kota	7	0	5	0	2	0	0	5	2
	**%**	**0.0**	**71.4**	**0.0**	**28.5**	**0.0**	**0.0**	**71.4**	**28.5**
Jhunjhunu	1	0	1	0	0	0	0	0	1
	**%**	**0.0**	**100.0**	**0.0**	**0.0**	**0.0**	**0.0**	**0.0**	**100.0**
Other	8	1	7	0	0	0	5	0	3
	**%**	**12.5**	**87.5**	**0.0**	**0.0**	**0.0**	**62.5**	**0.0**	**37.5**
**Total**	**291**	**15**	**236**	**25**	**15**	**45**	**33**	**68**	**145**
	**%**	**5.1**	**81.1**	**8.6**	**5.1**	**15.4**	**11.3**	**23.4**	**49.8**

### Omicron prevalence dynamics and clinical outcome

**Figure 1:**
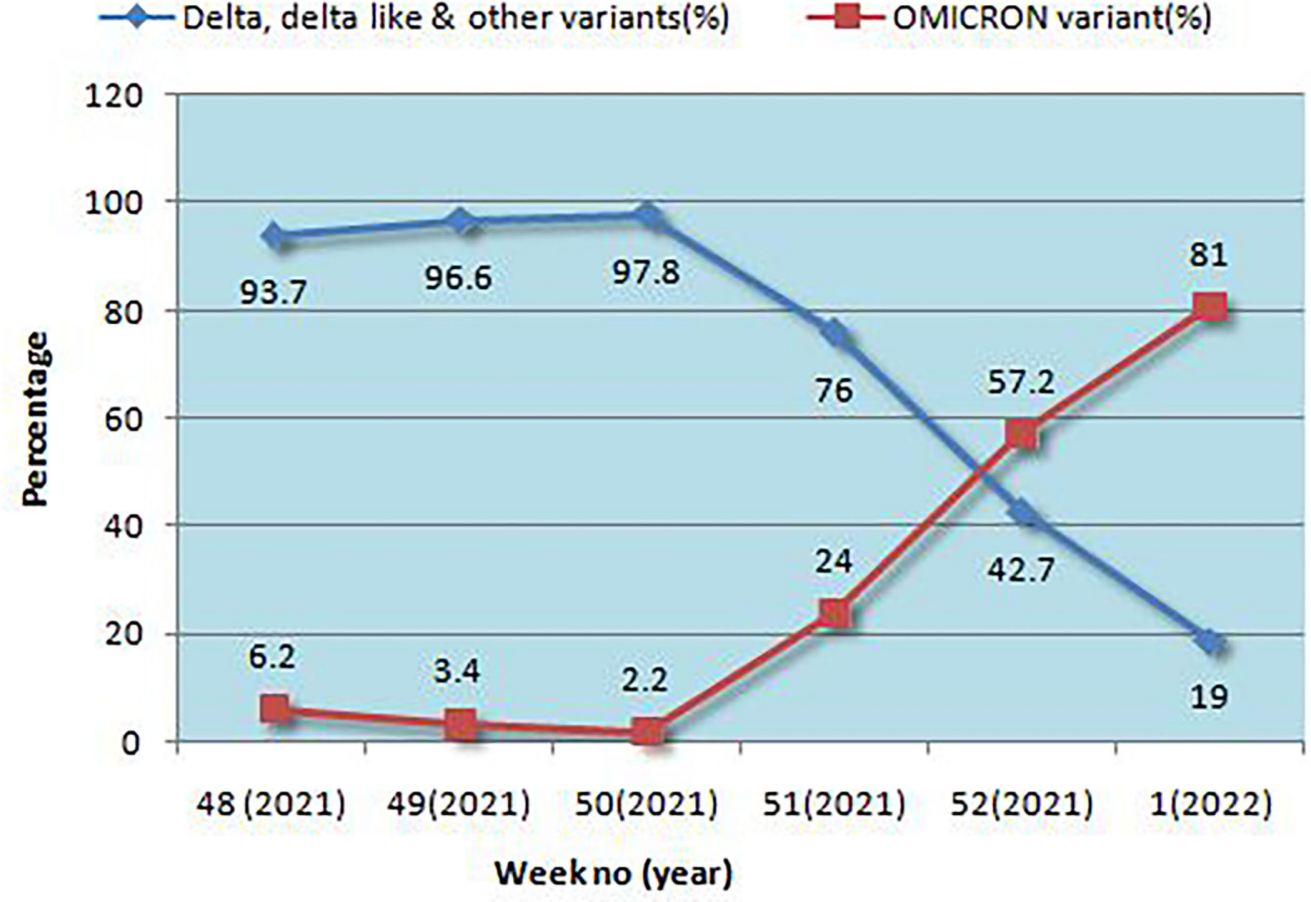
Week wise PCR positivity in Omicron and Delta cases.

Positivity for Omicron and other variants is given in Figure 1. In a very short time, Omicron rose from 6.2 to 81%, overtaking Delta and Delta-like variants. Initially, the B.1.1.529 lineage was 100% but later both BA.1 and BA.2 emerged, and BA.1 was the predominant lineage but BA.2 was found to be increasing each week. The highest positivity for Omicron was found in the Jaipur district followed by Ajmer and Udaipur (Table 1).

The clinical characteristics and outcomes of the patients included in this study are given in Table 2. Indicators of disease severity were more common in the Delta cases as compared to the Omicron cases, including hospitalization (8.92 vs. 1.03%) and O_2_ requirement (7.22 vs. 0.69%).

**Table 2:** Clinical outcome in Omicron and Delta cases.

**Patient details**	**Omicron** **(*N =* 291) (%)**	**Delta/Delta like/other variant** **(*N =* 762)**	**p-value**
**Clinical profile**			*P* < 0.00001
Asymptomatic	165 (56.70)	115 (15.09)	
Mild symptoms	97 (33.33)	356 (46.72)	
Moderate symptoms	29 (9.97)	291 (38.19)	
**Vaccination history**			*P* < 0.00001
Vaccinated	251 (86.25)	575 (75.50)	
Unvaccinated	15 (5.15)	187 (24.50)	
**Clinical outcome**			
Hospital admission	3 (1.03)	68 (8.92)	*P* < 0.0001
Oxygen requirement	2 (0.69)	55 (7.22)	*P* < 0.0001
Death	1 (0.34)	7 (0.92)	*P* = 0.45

**Figure 2:**
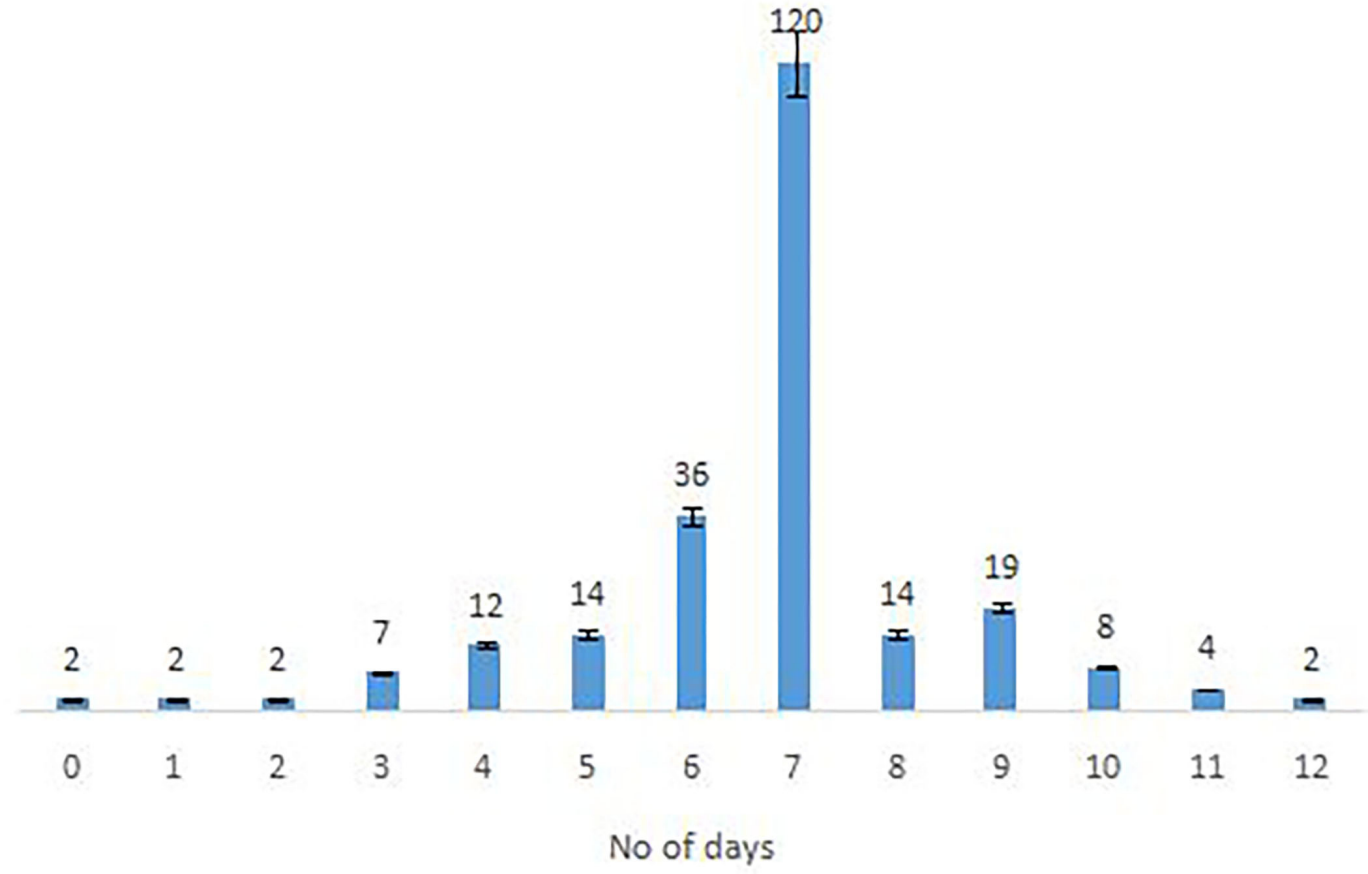
Time to recovery after PCR positivity in Omicron cases.

The majority of the Omicron cases were asymptomatic (56.7%), 33% had mild disease (sore throat and myalgia), and 9.6% had moderate disease/symptoms (fever, myalgia, cough, loss of taste, smell, etc.). Two patients >60 years of age with multiple co-morbidities who developed respiratory distress were hospitalized and required oxygen. One of them had recovered while the other died 7 days after illness. Metadata of the cases included in the study are given in Supplementary Table S1. Facility quarantine was done for Omicron-positive international travelers and their Omicron-positive contacts while other patients were home isolated. Time to recovery ranged from 0 to 15 days, the majority of patients recovered in 7 days' time (Figure 2), and only 1 (0.3%) patient died while the rest 290 (99.7%) patients recovered.

### Genomic analysis

**Figure 3:**
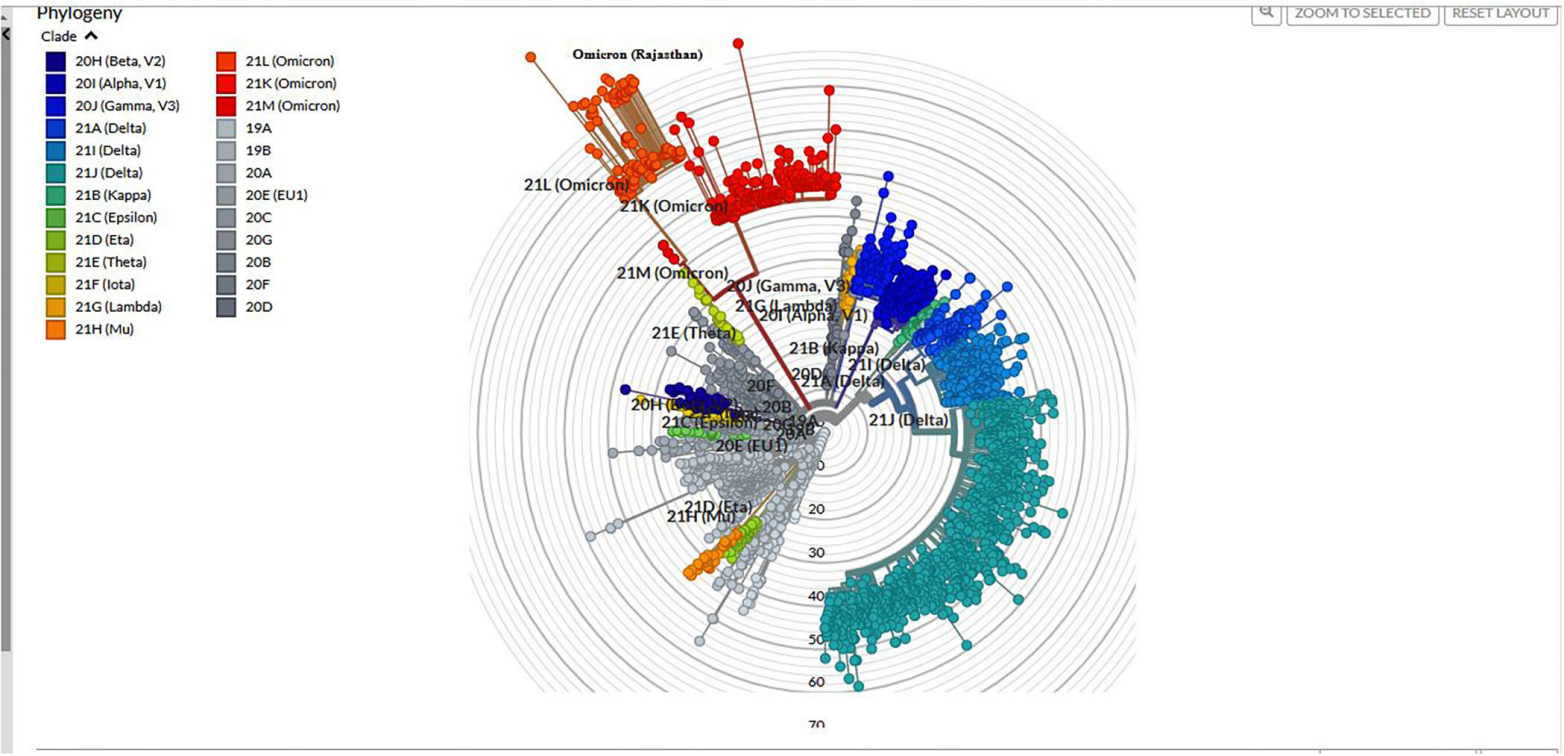
Phylogenetic tree of Omicron built using the Nextclade online tool.

Among 1,210 samples tested, 157 (12.9%) samples gave invalid results (>50%N) or were repeat samples (were removed from analysis). On Pangolin lineage analysis (accessed on 17 January 2022) of 1,053 (87%) samples, 762 (72.30%) belonged to Delta/Delta-like and other lineages [Delta (218; 20.7%), Delta-like (538; 51.1%), no VOC (4; 0.3%), Alpha (1; 0.1%), and B.1 (1; 0.1%)] and 291 (27.6%) were Omicron, out of which 13.40% were B.1.1.529, 62.88% were BA.1, and 23.72% were BA.2. The phylogenetic tree of Omicron cases is shown in Figure 3.

On re-analysis of 262 Omicron sequences (with >98% genome coverage) for mutation profiling, the lineage assignment was found to be BA.1 (***n*** = 106, 36.42%), BA.1.1 (***n*** = 44, 15.12%), and BA.2 (***n*** = 65, 22.33%) using the Pangolin online software (accessed on 02 June 2022). Sequences with <98% (***n*** = 30) genome coverage were not used for further analysis. Of the 262 sequences, Pangolin software has assigned the lineage to only 215 sequences, whereas 47 (16.15%) sequences were reported as unassigned (mixed lineage of Omicron).

In BA.1 and sub-lineages, 73% of sequences showed unique T1822I mutation in the ORF1ab region at nucleotide position 5,730. Surprisingly, spike gene relapse with the VOC mutation K417N, highly infectious variant N440K of SARS-CoV-2 Delta, and G446S mutation in receptor-binding domain (RBD) region of Omicron (B.1.1.529) was found in all BA.1 sequences. In 87% of sequences, the N211K mutation was found instead of N211I, the signature mutation of BA.1. Similarly, L212C mutation in the spike gene was found in 87% of sequences. In BA.1.1 Pangolin lineage, the spike region showed relapse mutation at K417N in all 44 sequences while mutation at N211K and L212C was observed in 84% of sequences, which are not signature mutations of BA.1.1 lineage (Table 3).

**Table 3:** Unique mutations detected in Omicron cases.

**Position**	**Mutations found**	**Absent in**	**Present in**
**BA.1 and derivatives (total = 106)**			
5730	T1822I	28	78
22813	K417N	0	106
22882	N440K	0	106
22898	G446S	0	106
22195	N211K (N211I is signature mutation)	13	93
22197	L212C	13	93
**BA.1.1 and derivatives (total = 44)**			
21846	T95I	29	15
22195	N211K	7	37
22197	L212C	7	37
22813	K417N	0	44
22882	N440K	1	43
	**BA.2 and derivatives (total = 65)**		
	**Unassigned (total = 35)**		

**Figure 4:**
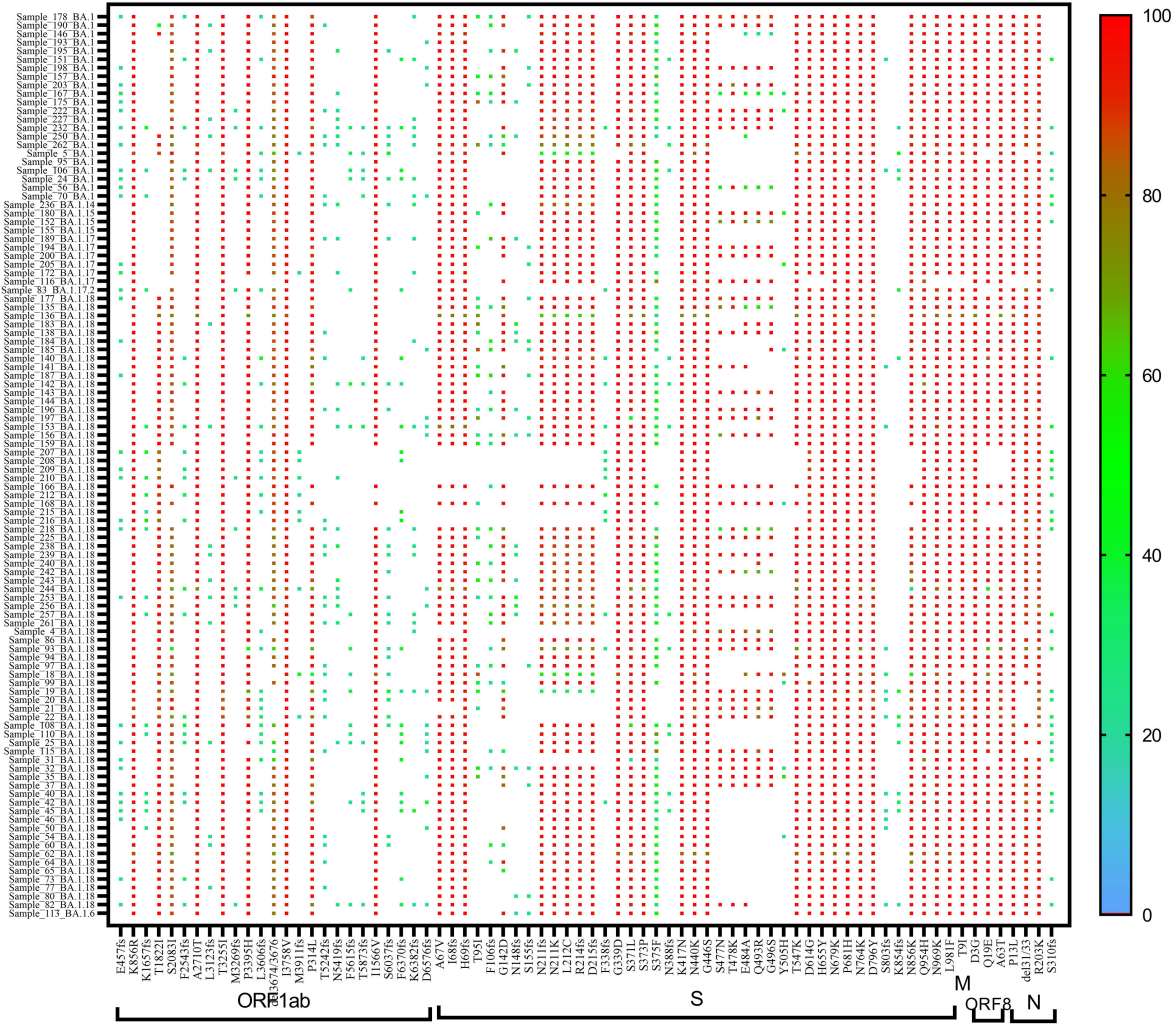
Heat map of Omicron (BA.1 and derivative) lineages.

**Figure 5:**
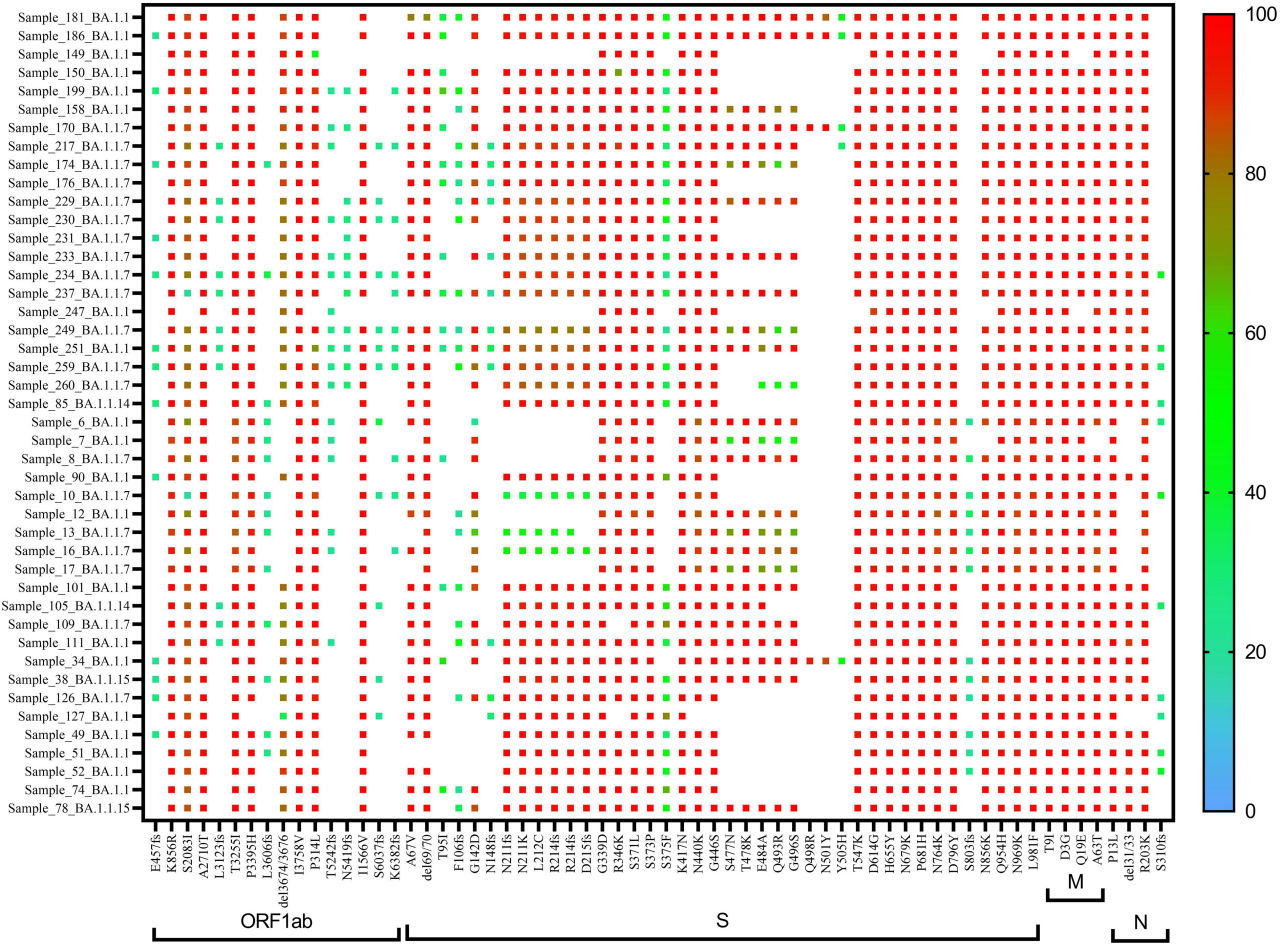
Heat map of Omicron (BA.1.1) lineage.

**Figure 6:**
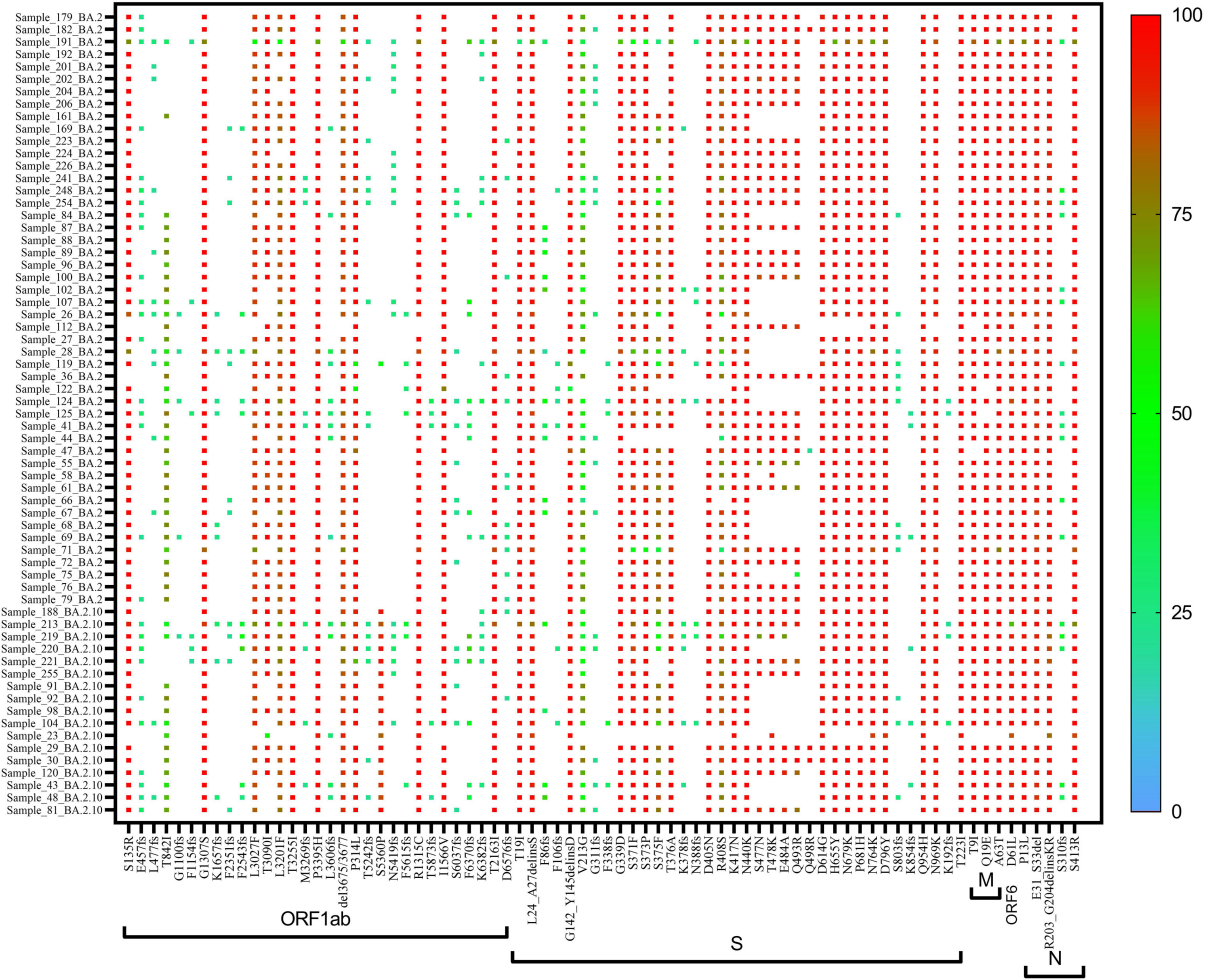
Heat map of Omicron (BA.2 and derivative) lineages.

A heat map generated using GraphPad software (PRISM 9.2.0) marked with all the signature mutations of each lineage and sub-lineages of BA.1, BA.1.1, and BA.2 is shown in Figures 4–6.

### Correlation of clinical profile with genomic analysis

The lineage detected in asymptomatic Omicron cases was BA.1–31.1%, BA.2–13.4%, and B1.1.529–12%. In cases with mild symptoms, the lineage detected was BA.1–23%, BA.2–9.2%, and B1.1.529–0.7%; in moderate symptom cases, BA.1 was 7.9%, BA.2 was 1%, and B.1.1.529 was 0.7%, both the two severe disease cases belonged to BA.1. Since BA.1 was the predominant lineage, it was found to be predominant among all the clinical groups up to January 2022. A total of three cases were found with severe symptoms, hospitalized, and were on oxygen support. On mutational analysis, we found that N211K was absent in all three cases. On mutational analysis of one death case out of the three hospitalized patients, it was found that T95I and K417N (nucleotide positions 21,846 and 22,813, respectively), mutations were found. However, in the other two cases, T1822I and N440K mutations (nucleotide positions 5,730 and 22,882, respectively) were also found along with the mutations mentioned above.

### Vaccination status of Omicron cases

**Figure 7:**
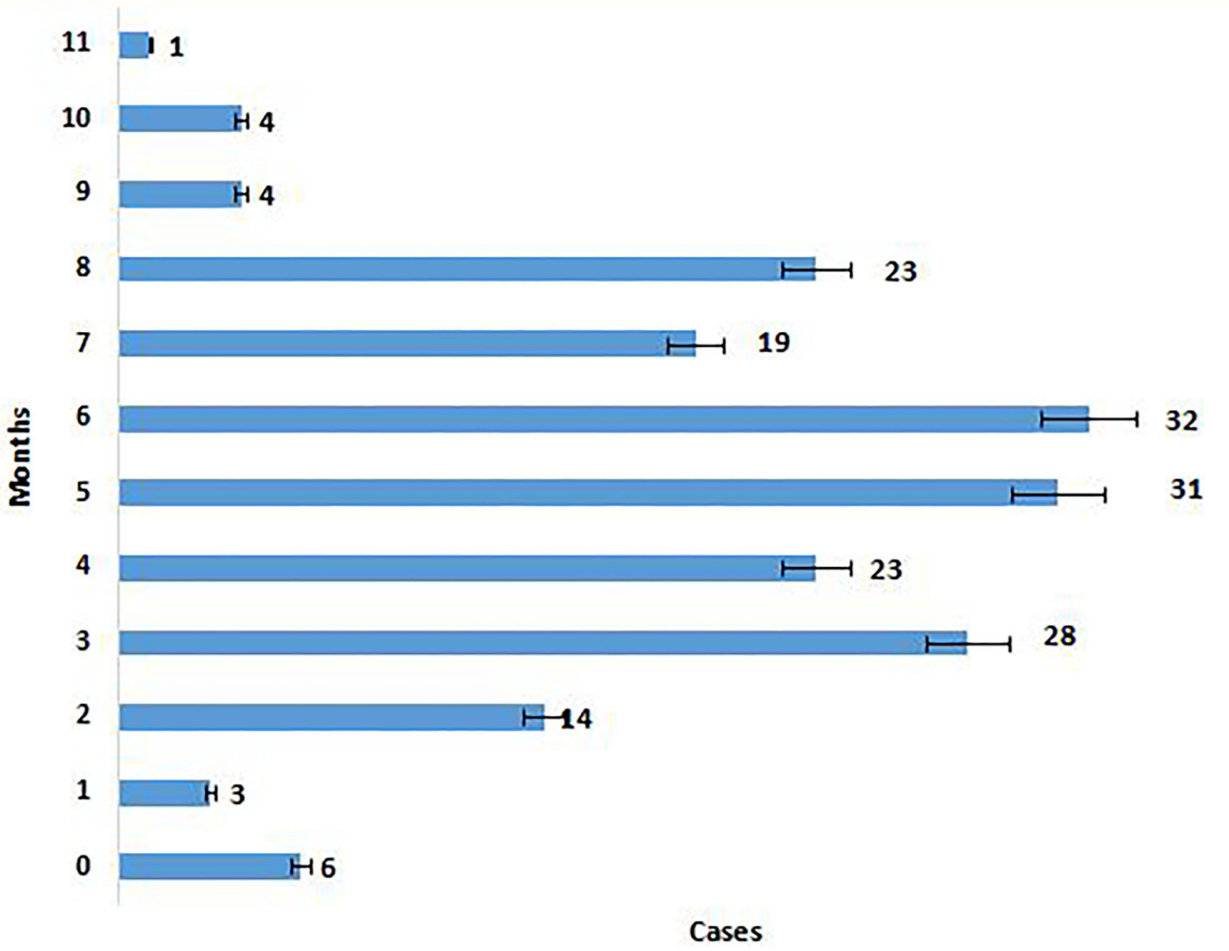
Months elapsed since vaccination and PCR positivity.

Details of vaccination status in various districts are given in Table 4, 81.1% were fully vaccinated and 5.1% had only one dose, 5.1% were not vaccinated, and 8.6% were not eligible for vaccination. Time elapsed between vaccination and RT-PCR positivity is given in Figure 7, in the majority (77.3%) of cases, it was <6 months. The majority (70.3%) of the patients had taken Covishield, 20.9% had taken Covaxin, 6.6% had taken Pfizer, 2.2% had taken Astra Zeneca, and 43.2% of the patients had a history of past infection in the last 6 months (Table 4).

**Table 4:** Details of travel and vaccination history in Omicron cases in Rajasthan.

**Country**	**Cases**	**%**
South Africa	5	11.1%
Ukraine	1	2.2%
Ghana	1	2.2%
Nigeria	4	8.9%
Zambia	2	4.4%
USA	7	15.6%
Tanzania	1	2.2%
UAE	11	24.4%
Spain	1	2.2%
UK	4	8.9%
Switzerland	1	2.2%
Congo	2	4.4%
London	1	2.2%
Italy	1	2.2%
Bangkok	1	2.2%
New York	1	2.2%
France	1	2.2%
Total	45	100.0%

## Discussion

Severe Acute Respiratory Syndrome-Coronavirus 2 (SARS-CoV-2) was first isolated from Wuhan, China in December 2019 (8). Soon the virus spread worldwide, and the pandemic was declared by WHO in March 2020. Since then, due to many mutations, different variants have emerged and caused multiple waves of infection. The second pandemic peak in India was caused by the Delta variant, which caused high morbidity and mortality, an increase in the number of hospitalizations, a high requirement for oxygen, and many cases of mucormycosis too. With the emergence of Omicron in South Africa, the concern was its effect on the Indian population. Initial cases were of B.1.1.529 lineage while in later contacts, BA.1 lineage emerged (9). In our study, only 15.4% had a history of international and 11.3% traveled to India, 23.4% were their contacts; but most importantly, 49.8% of cases had no history of travel or contact and this indicated that community transmission had occurred by late December 2021 to early January 2022. Even another study from Delhi reported community transmission by early January (10). At the same time, a rapid rise in infection was seen in Mumbai and Delhi (11). Ranjan reported that more than 50 countries including the United Kingdom, the United States, France, Italy, Netherlands, and India have caused new waves of Omicron (12).

In our study, the majority of patients were asymptomatic (56.7%) or had mild disease (33%), only 28 (9.6%) had moderate disease, and only two (0.7%) patients had severe disease requiring hospitalization. No difference was found in the clinical profile of different lineages of Omicron. These results are in concordance with another study from India (10). The majority of patients had recovered in 7 days' time except for one person who succumbed to death. Though initially, the international travelers and their contacts were admitted to designated areas of the hospital as part of facility quarantine. A recent study from Gauteng, South Africa reported that only 4.9% of cases were admitted to the hospital in the fourth wave (due to Omicron variant) as compared to 18.9 and 13.7% in the third and second waves (due to Delta and other variants). In addition, the study reported that 28.8% of admitted cases in the fourth wave had severe disease as compared to 60.1% and 66.9% in the third and second waves. As per the South African study, the proportion of cases admitted in the Omicron-dominated wave was lower than Delta-dominated wave and the severe cases were lower too (9, 13). However, a study from Imperial College London reports that there is no evidence that the disease severity or hospitalization due to Omicron is lower than Delta (13). Many factors, such as age, geographic area, and immunization coverage, can affect the severity of the disease. Peak positivity in Rajasthan during Delta-dominated second pandemic peak (May 2021) was 17.7% (341,957/1,926,446) with a 1.2% death rate (4,146/341,957) vs. 15.43% (9,016,687/58,454,872) positivity and 1.33% (120,072/9,016,687) death rates in India. The positivity during the third Omicron-dominated pandemic peak (January 2022) was 14.6% (250,194/1,706,003) and only 0.12% (304/250,194) death rate in Rajasthan vs. 12.77% (6,605,694/51,708,083) positivity and 0.22% (14,757/6,605,694) death rate in India. Vaccination plays a major role in preventing infections and in reducing the severity of the disease. As on 31 December 2021, the vaccination coverage was 35.02 million (with two doses administered) in Rajasthan and 606.2 million doses in India (IDSP, Jaipur Data). Not only the vaccination but previous infection also plays a role in reducing disease incidence, hospitalizations, and deaths. Moreover, there is a need for close monitoring of all hospitalized patients, especially severe cases and deaths during the Omicron-driven wave in India to understand the clinical and public health implications of the new variant. Omicron is reported to be more transmissible than Delta (2); the concern is that there is higher positivity due to Omicron than Delta. The Omicron poses a higher risk of re-infection than Delta (14) and the distribution by age, region, and ethnicity may also be different in Omicron (13). Various modeling groups have predicted that SARS-CoV-2 infections will reach an unprecedented peak in the next 1–2 months and may reach 35 million per day, which is triple the delta wave. It is estimated that the infection-hospitalization rate maybe 90–96% lower for Omicron as compared to Delta, and the infection-fatality rate will also be 97–99% lower for the same (15).^2^^,^^3^ Omicron has affected more than 140 countries worldwide and most of the states in India. The number of Omicron has also risen to more than 10,000 cases. India reported 285,000 new COVID-19 cases and 665 deaths in the last 24 h on 26 January 2022 with 2.223 million active cases; the daily positivity rate was 16.16% with a 93.2% recovery rate (16). Positivity was seen even when India has already administered 1,635.8 million vaccine doses till 26 January 2022. In a vast country, such as India, if only a small percentage of cases get hospitalized and few die, the sheer numbers get high and affect the health care systems. Since the majority of patients were asymptomatic or had mild disease, it was difficult to track the positive persons leading to the widespread in the community (2).

In the present study, K417N mutation was found in BA.1 and BA.1.1 sequences, which is a significant mutation contributing to the immune escape and higher infectivity as also reported in earlier studies (17–19). Other mutations responsible for immune escape, such as G446S and N440K, were also found in the sequences included in our study. Surprisingly, L212C mutation instead of L212I and N211K instead of N211I, the signature mutation of BA.1 was found in our sequences. We found that 23.7% of strains belonged to BA.2. This lineage does not have the SH69 and Sv70 deletions, which lead to S gene drop out or SGTF, which has been used as a proxy marker for Omicron in TaqPath PCR Kits, therefore, using such kits may not detect Omicron, which was also reported by Cobar and Cobar (1). Moreover, a rising trend in BA.2 lineage was found in our samples, hence use of these kits can give false negative for Omicron^4^ (20). However, a new kit Omisure (TATA Medical and Diagnostics) validated by the Indian Council of Medical Research (ICMR) for Omicron detection may be used to detect Omicron. This kit is particularly useful to identify if the hospitalized patient is Omicron or not, as with the increase in positivity, all the positives cannot be sequenced anymore (19)^5^.

We observed that 81.1% of patients were fully vaccinated and 5.1% were partially vaccinated, and the time elapsed in 77.3% of cases was <6 months. The majority (70.3%) of patients had taken Covishield, 20.6% had taken Covaxin while only a few had taken other vaccines abroad [Pfizer (6.6%) and AstraZeneca (2.2%)]. As per a serosurvey done in Rajasthan during the period November 2021–December 2021, 85–94% population had neutralizing antibodies against SARS-CoV-2 and even the unvaccinated had seropositivity of 74–84% cases depending on the dosage of vaccination taken (IDSP, Jaipur data). The low severity of diseases in these cases may be due to the protective effect of the past vaccination or infection. A study by Murhekar et al. reported a seroprevalence of 81 and 89.8%, among individuals who had received first and second vaccine doses, respectively, as compared to 62.3% in unvaccinated adults (21, 22). However, Omicron has been reported to evade the immune response both due to vaccination and due to earlier infection too (23–26). As per a report from Imperial College London, the vaccine effectiveness against the Omicron vs. Delta variant after two doses of vaccine (AstraZeneca or Pfizer) was 0% and 20%, and after the booster dose was 55% and 80%, respectively (13). As per the vaccine surveillance report from the UK, AstraZeneca vaccine efficacy after two doses against the Omicron was initially 45–50% but dropped to no effect after 20 weeks of the second dose. Similarly, after two doses of Moderna or Pfizer, the efficacy reduced from 65 to 10% by 25 weeks after the second dose. However, after the additional booster dose, the efficacy dropped from 65 to 25–40% in 15 weeks. Efficacy was better in the younger age group than in elder age and better in Delta than Omicron (27). An interesting observation in a study done at NIV, Pune demonstrated that substantial immune response was seen after breakthrough infection of Omicron against other variants. The sera of Omicron-infected persons could neutralize not only the Omicron but also other variants of concern, i.e., the most prevalent Delta variant, thus reducing the chances of reinfection due to Delta; hence replacing the Delta variant in the population. This stresses the urgent need to have an Omicron-specific vaccine strategy (28). Though the virus is known to evade the immune response, the severity of infection will be lower in the immunized person. Therefore, it is important to take booster/precautionary dose timely. Moreover, it is suspected that nonvaccinated may bear the brunt, so aggressive drives should be there to vaccinate all.

As per our preliminary data, the Omicron was found to be highly transmissible. In a very short time, it has spread in the community and has overtaken the existing Delta strain. It causes mainly asymptomatic to mild disease in vaccinated persons and severe disease in persons with co-morbidities. It is important to plan for Omicron-specific vaccination and give additional booster dose/precautionary doses to frontline workers and those with comorbidities on priority, and carry out sequencing of hospitalized, dead, and unvaccinated cases to know the variant responsible for the serious and unvaccinated cases.

## Limitation of the study

The samples included in our study were collected within a time frame of 2 months when there was a rise in Omicron cases as compared to Delta cases, which were decreasing each week. The comparison in the disease severity due to Delta is not truly reflected in our study as the morbidity and mortality due to Delta variant during the second pandemic peak, which was due to Delta, was much higher than that observed in our study. As a result, it reduces the significance of the comparison made between the two variants. A study comparing both variants during a longer period of time involving both waves would give a better picture.

## Data availability statement

The data presented in the study are deposited in the GISAID repository, accession numbers are provided in the supplementary data table 1.

## Ethics statement

The studies involving human participants were reviewed and approved by Ethics Committee, SMS Medical College and attached Hospitals Jaipur. Written informed consent from the participants' legal guardian/next of kin was not required to participate in this study in accordance with the national and the institutional requirements.

## Author contributions

SG, PS, FD, and DP carried out the experiments. AK, YJ, PP, SG, PS, BM, VP, and PY carried out the bioinformatics analysis. RS, RPS, and HS carried out data acquisition and compiling of data. NP, NB, and HS carried out data compilation. SB and NG carried out funding acquisition and review of manuscript and data. BM carried out conceptualization, manuscript preparation, data analysis, funding acquisition, and coordination of the research. RPS, RS, SG, and PS edited the manuscript. All authors have read and agreed to the published version of the manuscript.

## Funding

Financial support was provided by the Government of Rajasthan and Department of Health Research, Ministry of Health & Family Welfare, New Delhi to SMS Medical College VRDL, Jaipur.

## Conflict of interest

The authors declare that the research was conducted in the absence of any commercial or financial relationships that could be construed as a potential conflict of interest.

## Publisher's note

All claims expressed in this article are solely those of the authors and do not necessarily represent those of their affiliated organizations, or those of the publisher, the editors and the reviewers. Any product that may be evaluated in this article, or claim that may be made by its manufacturer, is not guaranteed or endorsed by the publisher.

## Footnotes

^1^https://www.cdc.gov/coronavirus/2019-ncov/variants/omicron-variant.html (accessed 29 Jan, 2022).

^2^ https://cmmid.github.io/topics/covid19/reports/omicron_england/report_11_dec_2021.pdf

^3^https://www.gov.scot/binaries/content/documents/govscot/publications/research-and-analysis/2021/12/omicron-scotland-evidence-paper/documents/omicron-scotland-evidence-paper/omicron-scotland-evidence-paper/govscot%3Adocument/omicron-scotland-evidence-paperexternalicon;https://indianexpress.com < India

^4^https://www.fhi.no/en/news/2021/updated-risk-assessment-about-omicron-variant/externalicon.

^5^https://www.icmr.gov.in/pdf/covid/kits/archive/RT_PCR_kits_for_OMICRON_VOC_30122021.pdf.

## Supplementary material

Supplementary Table 1 Metadata of Omicron, Delta, and other variants.

## References

[1] Cobar O Cobar S. Omicron S: 69-70 deletion and SARS-CoV-2 Detection Test Evasion. Literature Review. (2022). doi 10.13140/RG.2.2.21976.78088.

[2] Karim SSA Karim QA. Omicron SARS-CoV-2 variant: a new chapter in the COVID-19 pandemic. Lancet. (2021) 398:2126–8. 10.1016/S0140-6736(21)02758-6 34871545 PMC8640673

[3] Nishiura H Ito K Anzai A Kobayashi T Piantham C Rodríguez-Morales AJ. Relative reproduction number of SARS-CoV-2 omicron (B11529) compared with delta variant in South Africa. J Clin Med. (2022) 11:30. 10.3390/jcm11010030 35011781 PMC8745053

[4] Ontario Agency for Health Protection and Promotion (Public health Ontario). Omicron disease severity – what we know so far. Toronto, ON: Queen's Printer for Ontario; 2022.

[5] Sy KTL White LF Nichols BE. Population density and basic reproductive number of COVID-19 across United States counties. PLoS ONE. (2021) 16:e0249271. 10.1371/journal.pone.0249271 33882054 PMC8059825

[6] Application note. Infectious disease research “Highly sensitive detection of SARS-CoV-2variants with the Ion GeneStudio S5 System”. Available online at: ThermofischerScientific.com (accessed November 26, 2021).

[7] Global Initiative on Sharing All Influenza Data (GISAID). 2020 Clade and lineage nomenclature aids in genomic epidemiology studies of active hCoV-19 viruses. Available online at: https://www.gisaid.org/references/statements-clarifications/clade-and-lineage-nomenclature-aids-in-genomic-epidemiology-of-active-hcov-19-viruses/ (accessed February 02, 2022).

[8] Wu F Zhao S Yu B Chen YM Wang W Song ZG. A new coronavirus associated with human respiratory disease in China. Nature. (2020) 579:265–9. 10.1038/s41586-020-2008-3 32015508 PMC7094943

[9] Potdar VA Yadav P Lole K Cherian S Shastri J Malhotra B. Detection of the omicron variant in international travellers and their family contacts in India. medRxi v. (2021). 10.1101/2021.12.27.21268429

[10] Garg R Gautam P Suroliya V Agarwal R Bhugra A Kaur US. Evidence of early community transmission of Omicron (B1. 1529) in Delhi- A city with very high seropositivity and past-exposure! Travel. Med Infect Dis. (2022) 46:102276. 10.1016/j.tmaid.2022.102276 35181557 PMC9759830

[11] The Times of India. Omicron variant live updates: Mumbai, Delhi, Gujarat report big spike in COVID cases. Available online at: https://timesofndia.indiatimes.com/india/covid-vaccination-omicronvariant-india-live-updates-28-december-2021/liveblog/88532102. cms. (accessed on 29 Dec, 2021).

[12] Ranjan R. Omicron impact in india: an early analysis of the ongoing covid-19 third wave. MedRxiv. (2022). 10.1101/2022.01.09.22268969

[13] Waasila J Karim SA Mudara C Welch R Ozougwu L Groome MJ. Clinical severity of COVID-19 patients admitted to hospitals in Gauteng, South Africa during the omicron-dominant fourth wave. Lancet. (2022) 10:E961–9. 10.1016/S2214-109X(22)00114-0 35597249 PMC9116895

[14] Ferguson N Ghani A Cori A Hogan A Hinsley W Volz E. Growth, Population Distribution and Immune Escape of the Omicron in England. Imperial College London. (2021).

[15] Mohsin Md Sultan M. Omicron SARS-CoV-2 variant of concern. A review on its transmissibility, immune evasion, reinfection, and severity. Medicine. (2022) 101:p e29165. 10.1097/MD.0000000000029165 35583528 PMC9276130

[16] Khandia R Singhal S Alqahtani T Kamal MA El-Shall NA Nainu F. Emergence of SARS-CoV-2 omicron (B. 11529) variant, salient features, high global health concerns and strategies to counter it amid ongoing COVID-19 pandemic. Environ Res. (2022) 209:112816. 10.1016/j.envres.2022.112816 35093310 PMC8798788

[17] Bansal K Kumar S. Mutational cascade of SARS-CoV-2 leading to evolution and emergence of omicron variant. Virus Res. (2022) 2:315:198765. 10.1016/j.virusres.2022.198765 35367284 PMC8968180

[18] Kumar S Bansal K. Cross-sectional genomic perspective of epidemic waves of SARS-CoV-2: a pan India study. Virus Res. (2022) 308:198642. 10.1016/j.virusres.2021.198642 34822953 PMC8606321

[19] Thakur V Ratho RK OMICRON. (B. 11529): A new SARS-CoV-2 variant of concern mounting worldwide fear. J Med Virol. (2022) 94:1821–4. 10.1002/jmv.27541 34936120

[20] SARS-CoV-2 Viral Mutations: Impact on COVID-19 Tests. FDA. Available online at: https://www.fda.gov/medical-devices/coronavirus-covid-19-and-medical-devices/SARS-CoV-2-viral-mutations-impact-covid-19-tests#taqpath (accessed May 20, 2022).

[21] Murhekar MV Bhatnagar T Selvaraju S Saravanakumar V Thangaraj JWV Shah N et al. SARS-CoV-2 antibody seroprevalence in India, August-September, 2020: findings from the second nationwide household serosurvey. Lancet Glob Health. (2021) 9:e257–66. 10.1016/S2214-109X(20)30544-1 33515512 PMC7906675

[22] Mills MG Hajian P Bakhash SM Xie H Mantzke D Zhu H et al. Rapid and accurate identification of SARS-CoV-2 omicron variants using droplet digital PCR (RT-ddPCR). J Clin Virol. (2022) 154:105218. 10.1016/j.jcv.2022.105218 35779343 PMC9212762

[23] Liu L Iketani S Guo Y Chan JF Wang M Liu L et al. Striking antibody evasion manifested by the Omicron variant of SARS-CoV-2. Nature. (2022) 602:676–81. 10.1038/s41586-021-04388-0 35016198

[24] Nikhra Vinod. (2021). Date with the Pandemic - Vinod Nikhra M.D. Book - Paperback and Hard cover. Available online at: Amazon.com.

[25] Nikhra V. Projections for COVID-19 in Present Context: Journey of the Pandemic to Endemicity. EC Microbiology. (2022) 19–26.

[26] Zhang L Li Q Liang Z Li T Liu S Cui Q. The significant immune escape of pseudotyped SARS-CoV-2 variant Omicron. Emerg Microbes Infect. (2022): 11:1–5. 10.1080/22221751.2021.2017757 34890524 PMC8725892

[27] COVID-19 vaccine surveillance report Week 427 January 2022. UKHealthsecurity agency. Available online at: https://assets.publishing.service.gov.uk/government/uploads/system/uploads/attachmentdata/file/1050721/Vaccine-surveillance-report-week-4.pdf (accessed May 23, 2022).

[28] Yadav PD Sapkal G Sahay RR Potdar V Deshpande G Patil DY. Substantial immune response in Omicron infected breakthrough and unvaccinated individuals against SARS-CoV-2 variants of concerns. J Infect. (2022) 84:e80–1. 10.1016/j.jinf.2022.02.005 35157945 PMC8837481

